# A practical illustration of spatial smoothing methods for disconnected regions with INLA: spatial survey on overweight and obesity in Malaysia

**DOI:** 10.1186/s12942-023-00336-5

**Published:** 2023-06-21

**Authors:** Maria Safura Mohamad, Khairul Nizam Abdul Maulud, Christel Faes

**Affiliations:** 1 Faculty of Social Sciences, Unit of Health Sciences, Tampere University, Arvo Ylpön Katu 34, 33520 Tampere, Finland; 2 Department of Civil Engineering, Faculty of Engineering & Built Environment, National University of Malaysia, 43600 Bangi, Selangor Malaysia; 3 Earth Observation Centre, Institute of Climate Change, National University of Malaysia, 43600 Bangi, Selangor Malaysia; 4 Data Science Institute, I-BioStat, Hasselt University, Martelarenlaan 42, 3500 Hasselt, Belgium

**Keywords:** Bayesian hierarchical modelling, Children, Disconnected regions, Disease mapping, INLA, Malaysia, Obesity, Overweight

## Abstract

**Background:**

National prevalence could mask subnational heterogeneity in disease occurrence, and disease mapping is an important tool to illustrate the spatial pattern of disease. However, there is limited information on techniques for the specification of conditional autoregressive models in disease mapping involving disconnected regions. This study explores available techniques for producing district-level prevalence estimates for disconnected regions, using as an example childhood overweight in Malaysia, which consists of the Peninsular and Borneo regions separated by the South China Sea. We used data from Malaysia National Health and Morbidity Survey conducted in 2015. We adopted Bayesian hierarchical modelling using the integrated nested Laplace approximation (INLA) program in R-software to model the spatial distribution of overweight among 6301 children aged 5–17 years across 144 districts located in two disconnected regions. We illustrate different types of spatial models for prevalence mapping across disconnected regions, taking into account the survey design and adjusting for district-level demographic and socioeconomic covariates.

**Results:**

The spatial model with split random effects and a common intercept has the lowest Deviance and Watanabe Information Criteria. There was evidence of a spatial pattern in the prevalence of childhood overweight across districts. An increasing trend in smoothed prevalence of overweight was observed when moving from the east to the west of the Peninsular and Borneo regions. The proportion of Bumiputera ethnicity in the district had a significant negative association with childhood overweight: the higher the proportion of Bumiputera ethnicity in the district, the lower the prevalence of childhood overweight.

**Conclusion:**

This study illustrates different available techniques for mapping prevalence across districts in disconnected regions using survey data. These techniques can be utilized to produce reliable subnational estimates for any areas that comprise of disconnected regions. Through the example, we learned that the best-fit model was the one that considered the separate variations of the individual regions. We discovered that the occurrence of childhood overweight in Malaysia followed a spatial pattern with an east–west gradient trend, and we identified districts with high prevalence of overweight. This information could help policy makers in making informed decisions for targeted public health interventions in high-risk areas.

**Supplementary Information:**

The online version contains supplementary material available at 10.1186/s12942-023-00336-5.

## Background

Health indicators that are routinely collected by national surveys are usually reported at the national level, including the prevalence of overweight and obesity. These are important indicators for monitoring trends and making comparisons between countries. Nevertheless, national averages could mask the fine geographical variations and inequalities regarding health indicators [1]. In addition, local stake holders have great need for reliable estimates at the subnational level, such as the district or sub-district level. This information can be used for ensuring equitable resource allocation, facilitating optimal local-level planning, and implementation of interventions and health programs [2]. Obtaining reliable estimates at these levels requires a large sample size that is representative of the population, and this could greatly increase the survey cost and time, which could be a burden in countries with limited resources. To overcome this issue, mapping disease prevalence has been widely used to identify spatial pattern of disease, identify areas with high disease burden, assist in disease surveillance, and understand the aetiology of diseases [3]. However, direct estimates of disease prevalence in an area based on samples available in the area could lead to unstable estimates.

To address this problem, Bayesian hierarchical modelling incorporating spatial smoothing has been commonly used in disease mapping to improve prevalence estimates. In Bayesian spatial analysis, information from a neighbouring area is used, and the mean of an area is assumed to be the average of neighbouring areas [4]. This “borrowing of information” helps to overcome issues of data sparseness and account for spatial dependency. In addition, complex sampling designs are often used in the collection of survey data and need to be accounted for in the analysis. Mercer et al. [5] and Vandendijck et al. [6] have reviewed methods for the analysis of spatial health surveys with a complex sampling design and possible missing data. They demonstrated that methods that considered sampling weight in the analysis performed well in simulations and decreased non-response and selection bias [5, 6].

In disease mapping, the spatial dependency between neighbouring areas needs to be defined prior to analysis. The intrinsic conditional autoregressive (ICAR) model is the most popular approach for specifying the spatial dependency [7]. When defining the ICAR model, it is necessary to specify a graph that consists of nodes and edges that represent the respective regions and neighbouring relationships between them [8]. In a connected graph, all pairs of nodes are connected, while a disconnected graph can arise when there are nodes with no neighbours or when the study region is split, resulting in separate subgraphs [8].

However, there is limited information on techniques for the specification of the ICAR model in disease mapping involving disconnected regions. In the present study, we propose an adapted Bayesian spatial model to deal with disconnected regions, which is later denoted as the split random effects model. We explore such techniques using as an example childhood overweight in Malaysia, which is geographically split into Peninsular and Borneo regions separated by the South China Sea. Studying this is important because worldwide, prevalence of overweight and obesity is increasing among children. If not prevented early, the condition can persist into adulthood and be associated with multiple chronic diseases and psychological disorders [9, 10]. In addition, premature mortality and productivity losses associated with the condition have impacts on a country’s economy from indirect costs [11]. The prevalence of overweight and obesity among adolescents in Malaysia is among the highest in Southeast Asia [12], and there is evidence of increasing trends over time [13]. However, the broader geographical distribution and areas that are most affected remain unclear. Therefore, the identification of vulnerable areas is vital to improve the prevention of childhood overweight and obesity.

The aims of the present study were to analyse the geographical variation of the estimated prevalence of overweight (including obesity) and to identify areas of unusually high prevalence at the district level. This analysis examined children aged 5–17 years in Malaysia using Bayesian hierarchical modelling. Malaysia is geographically split into two disconnected regions: Peninsular Malaysia and Borneo’s East Malaysia, which are separated by the South China Sea, and which makes the analysis more difficult. Models for producing district-level prevalence estimates for disconnected regions were explored.

## Methods

### Study design

We used cross-sectional population-based survey data from the National Health and Morbidity Survey (NHMS) conducted in 2015. The NHMS applied a two-stage stratified cluster sampling method to obtain a nationally representative sample of urban and rural populations. Malaysia was stratified into states including Federal Territories, followed by secondary strata, which consist of urban and rural strata formed within the primary stratum. In total, 30 urban and rural strata were created.

In the first sampling stage, 536 and 333 enumeration blocks (primary sampling units) were selected in proportion to the population sizes from urban and rural strata, respectively. The sampling frame of the enumeration blocks was provided by the Department of Statistics Malaysia. Each enumeration block comprises an average of 80 to 120 living quarters with an average population of 500 to 600 people. In the second sampling stage, 12 living quarters (secondary sampling units) were randomly selected from each selected enumeration block. A total of 10,428 living quarters were selected in this survey [14].

All households and their members within the selected living quarters were included in the study. The response rates were 89.2% and 96.9% at the living-quarter level and the individual level, respectively. The overall response rate was 86.4% [14]. The NHMS carried out face-to-face interviews using a pre-tested questionnaire. Children aged 13 years or older were interviewed directly, while parents or guardians provided responses by proxy for children younger than 13 years old. Physical measurements of height and weight were conducted by trained research assistants.

### Study area

Malaysia is situated in the Southeast Asia region at 4.1936° N, 103.7249° E [15]. It is split into two regions: Peninsular Malaysia, which covers the southernmost point of Eurasia, and Malaysian Borneo (East Malaysia), which is on the island of Borneo. Malaysia covers an area of 329,847 km^2^ (127,355 miles^2^) and consists of 16 states, including 3 federal territories and 144 administrative districts: 87 in the Peninsular and 57 in Borneo. The population was 31.2 million in 2015 [16].

### Dependent variable

In this study, information from children aged 5 to 17 years was used. The outcome variable was overweight (including obesity) based on measured height and weight. The children's body mass indexes (BMIs) were classified according to the age and sex-specific BMI criteria of the International Obesity Taskforce (IOTF) 2012, which uses cut-offs for BMI that correspond to adult BMI cut-offs of 25 at age 18 [17]. For each child who participated in the survey, the coordinates of the living quarter were recorded.

### District-level covariates

Table 1 shows several district-level covariates that were considered in our analysis. These include the proportion of district population aged 5–17 years, proportion of females, proportion of Bumiputera ethnicity, average household size, median gross monthly household income (in Malaysian ringgit), and population density (in km^2^). All district-level covariate information was obtained from the Department of Statistics Malaysia [18].

**Table 1:** District-level covariates used in our analysis of prevalence of overweight among Malaysian children. [18]

District-level covariates	Details
Proportion of children aged 5–17 years	A state-level proportion of 5–17 years old in 2015 was used for projection of district-level proportion, based on 2016 data
Proportion of females	District-level population and demography statistics 2016
Proportion of Bumiputera ethnicity	District-level population statistics by ethnicity 2016
Average household size	Population statistics 2016: Average household size by district
Median household income (Malaysian ringgit)	Household income and expenditure statistics 2016
Population density (square km)	Population statistics 2016: Population density by district

### Ethical considerations

NHMS 2015 received ethical approval from the Medical Research Ethics Committee, Ministry of Health Malaysia (NMRR–14-1064-21877). Written informed consent was obtained from each participant, including parents or guardians of the children. The present study was registered at the Malaysia National Medical Research Register, and approval for data usage was received from the Director General of Health, Malaysia.

## Statistical analysis

### Traditional spatial smoothing model

Using the design-based approach, district-level prevalence estimates of overweight can be obtained directly from the available samples in each district. However, in districts not included in NHMS 2015, no estimates can be obtained. In addition, estimates with large variances are inevitable in districts with sparse data. Therefore, we employed a fully Bayesian hierarchical modelling framework that allows spatial smoothing to obtain prevalence estimates in unsampled districts and to produce more reliable estimates with smaller variances.

We can assume a Bernoulli model for the binary response variable of overweight (${{\varvec{y}}}_{{\varvec{i}}{\varvec{j}}}=1$ if overweight versus ${{\varvec{y}}}_{{\varvec{i}}{\varvec{j}}}=0$ if non-overweight) for an individual child ${\varvec{i}}$ (${\varvec{i}}$ = 1, …, 6301) in administrative district ${\varvec{j}}$ (${\varvec{j}}$ = 1, …, 144) as follows:

1 $${{\varvec{y}}}_{{\varvec{i}}{\varvec{j}}\boldsymbol{ }}\sim \boldsymbol{ }{\varvec{B}}{\varvec{e}}{\varvec{r}}{\varvec{n}}{\varvec{o}}{\varvec{u}}{\varvec{l}}{\varvec{l}}{\varvec{i}}({{\varvec{p}}}_{{\varvec{i}}{\varvec{j}}})$$

2 $$\mathbf{l}\mathbf{o}\mathbf{g}\mathbf{i}\mathbf{t}({{\varvec{p}}}_{{\varvec{i}}{\varvec{j}}})=\boldsymbol{\alpha }+{\sum }_{{\varvec{k}}}{{\varvec{\beta}}}_{{\varvec{k}}}{{\varvec{x}}}_{{\varvec{j}}{\varvec{k}}}+{{\varvec{v}}}_{{\varvec{j}}}+{{\varvec{u}}}_{{\varvec{j}}}$$

In this model, ${{\varvec{p}}}_{{\varvec{i}}{\varvec{j}}}$ is the probability of the ${\varvec{i}}$ th child in the ${\varvec{j}}$ th district being overweight. The logit link function was applied to link the probability with the potential district-level covariates ${{\varvec{x}}}_{{\varvec{j}}}$ with associated parameters ${\varvec{\beta}}.$ The intercept $\boldsymbol{\alpha }$ represents the overall risk of being overweight.

This model-based approach allows for incorporation of random effects to account for the spatial correlation between districts, while the unstructured random effects allow each district to vary independently of its adjoining neighbours. We applied the Besag, York, and Mollié model, which partitions the random effects at the district level into unstructured (${{\varvec{v}}}_{{\varvec{j}}}$) and spatially structured (${{\varvec{u}}}_{{\varvec{j}}}$) effects [7]. We incorporated the ICAR prior in the spatially structured random effects, which has the following conditional distribution:

3 $${{\varvec{u}}}_{{\varvec{j}}}|{{\varvec{u}}}_{\left(-{\varvec{j}}\right)}\sim {\varvec{N}}\left(\frac{{\sum }_{{{\varvec{j}}}^{\mathbf{^{\prime}}}}{{\varvec{w}}}_{{\varvec{j}}{{\varvec{j}}}^{\mathbf{^{\prime}}}}{{\varvec{u}}}_{{{\varvec{j}}}^{\mathbf{^{\prime}}}}}{{\sum }_{{{\varvec{j}}}^{\mathbf{^{\prime}}}}{{\varvec{w}}}_{{\varvec{j}}{{\varvec{j}}}^{\mathbf{^{\prime}}}}},\frac{1}{{\varvec{\tau}}{\sum }_{{{\varvec{j}}}^{\mathbf{^{\prime}}}}{{\varvec{w}}}_{{\varvec{j}}{{\varvec{j}}}^{\mathbf{^{\prime}}}}}\right),$$

where ${\varvec{N}}$ denotes the normal distribution, and ${{\varvec{w}}}_{{\varvec{j}}{\varvec{j}}\mathbf{^{\prime}}}$ is a neighbourhood weight. We denote this as ${{\varvec{u}}}_{{\varvec{j}}}\sim {\varvec{C}}{\varvec{A}}{\varvec{R}}({\varvec{\tau}},{\varvec{W}})$, with ${\varvec{W}}={({\varvec{w}}}_{{\varvec{j}}{\varvec{j}}\mathbf{^{\prime}}})$. This ICAR prior allows us to borrow information between neighbouring areas, yielding a smoothed prevalence map. A scaled version of ${\varvec{W}}$ is recommended such that ${\varvec{\tau}}$ can be interpreted as the marginal precision [19].

### Accounting for the study design

In order to reduce bias due to non-random sampling and non-response, sampling weight was accounted for in all analyses. First, we computed the design-based Horvitz-Thompson estimator [20], ${\widehat{{\varvec{p}}}}_{{\varvec{j}}}$, which is the district-specific prevalence of overweight, using the observations in each district:

4 $${\widehat{{\varvec{p}}}}_{{\varvec{j}}\boldsymbol{ }}=\frac{\sum_{{\varvec{i}}}{{\varvec{y}}}_{{\varvec{i}}{\varvec{j}}\boldsymbol{ }}{{\varvec{s}}}_{{\varvec{i}}{\varvec{j}}\boldsymbol{ }}}{{{\varvec{s}}}_{{\varvec{i}}{\varvec{j}}}}$$

where ${{\varvec{s}}}_{{\varvec{i}}{\varvec{j}}}$ is the sampling weight of individual child ${\varvec{i}}$ in district ${\varvec{j}}$. This estimator takes into account the design of the study. We then obtain an area-level summary by applying the empirical logistic transformation of ${\widehat{{\varvec{p}}}}_{{\varvec{j}}}$, as described by Mercer et al. [5] in a study comparing different weighting methods when using spatial smoothing in small-area estimations (i.e. ${{{\varvec{y}}}_{{\varvec{j}}}}^{\mathrm{L}}={\varvec{l}}{\varvec{o}}{\varvec{g}}\left[\frac{{\widehat{{\varvec{p}}}}_{{\varvec{j}}}}{1-{\widehat{{\varvec{p}}}}_{{\varvec{j}}}}\right]$) [5]. We then model this summary data as:

5 $${\varvec{y}}_{{\varvec{j}}}^{L} |\user2{ p}_{{\user2{j }}} \user2{ }\sim \user2{ N}\left( {\user2{log }\left[ {\frac{{{\varvec{p}}_{{\user2{j }}} }}{{1 - {\varvec{p}}_{{\user2{j }}} }}} \right],\frac{{{\varvec{var}}(\left( {\hat{\user2{p}}_{{\user2{j }}} } \right)}}{{\hat{\user2{p}}_{j}^{2} \left( {1 - \hat{\user2{p}}_{{\user2{j }}} } \right)^{2} }}} \right)$$

where ${\varvec{v}}{\varvec{a}}{\varvec{r}}({\widehat{{\varvec{p}}}}_{{\varvec{j}}})$ is the variance of the Horvitz-Thompson estimator ${\widehat{{\varvec{p}}}}_{{\varvec{j}}}$ and ${{\varvec{p}}}_{{\varvec{j}}}$ from the previous section, which takes into account the study design in both the estimator and its variance.

### Bayesian inference

We performed the Bayesian analysis using an integrated nested Laplace approximation (INLA) program in R software [21]. The deterministic algorithm approach for Bayesian inference in INLA has been proven to reduce the computing time and provides accurate results [22, 23]. In Bayesian inference, prior distributions for parameters to be estimated were specified before modelling was commenced. In R-INLA, the default Gaussian prior with mean and precision equal to 0 was specified for the intercept of the model, **α**. For the fixed effects, Gaussian priors with mean equal to 0 and precision equal to 0.001 were assigned.

We specified the unstructured random effect as a normal distribution with a standardized mean of zero. A gamma distribution (0.5, 0.005) was specified for the hyperpriors for the precision of random effects. We report the covariate effects using the mean and 95% credible intervals, which represent the range of values that contains the true value with a probability of 95%.

To evaluate the model fit, the deviance information criterion (DIC) and Watanabe information criterion (WAIC) were used [24, 25]. Lower DIC or WAIC values signify better model fit. Once the best-fit model was identified, it was used to produce prevalence estimates for each district. District-level covariates were then introduced to the model individually. The model was built using a forward stepwise regression approach, and its performance was again assessed using the DIC and WAIC values.

### Proposed spatial models for disconnected regions

The Bayesian hierarchical spatial model uses neighbourhood weights that are traditionally defined as ${{\varvec{w}}}_{{\varvec{j}}{\varvec{j}}\mathbf{^{\prime}}}=1$ when areas ${\varvec{j}}$ and ${\varvec{j}}\mathbf{^{\prime}}$ share a boundary and as ${{\varvec{w}}}_{{\varvec{j}}{\varvec{j}}\mathbf{^{\prime}}}=0$ otherwise [26]. This definition defines a graph as a compilation of nodes and edges representing the respective districts and the set of neighbours for each district. Most often in disease mapping, we assume that the graph is a connected graph, meaning that all the nodes connect to at least one other node. However, disconnected graphs can arise when there is an island with no neighbour or when the study region is split, resulting in separate subgraphs [8].

Analysis involving disconnected subgraphs is not as straightforward as the analysis for a connected graph and is very rarely discussed. Hodges et al. [27] discuss the theory of setting disconnected subgraphs [27], while Freni-Sterrantino et al. [8] give some recommendations in INLA [8]. We give an overview of techniques available in INLA for producing district-level prevalence estimates for disconnected regions. The focus is on the setting of disconnected regions that consist of multiple areas ${\varvec{j}}$ (thus, there are no singletons).

We assume that the total study region with areas ${\varvec{j}}$ (e.g., districts) is split up into disconnected regions ${\varvec{r}}$. In the application presented in this paper, the South China Sea separates the region of Malaysia into two disconnected regions (Peninsular Malaysia and Borneo).

### Model I: single sum-to-zero constraint

The ICAR distribution has an improper distribution, and the standard method to deal with this is by adding a sum-to-zero constraint—i.e., the sum of all random effects is equal to zero. This is the standard method used in disease mapping with a connected graph. In the case of disconnected regions, this assumption can still be made. Using this assumption, the overall mean of the random effects across the whole study area is zero. The spatial random effects for area ${\varvec{j}}$ in region ${\varvec{r}}$, ${{\varvec{u}}}_{{\varvec{j}}({\varvec{r}})}$, can be interpreted as the area-specific deviation from the overall risk.

6 $${\varvec{l}}{\varvec{o}}{\varvec{g}}{\varvec{i}}{\varvec{t}}({{\varvec{p}}}_{{\varvec{i}}{\varvec{j}}({\varvec{r}})})=\boldsymbol{\alpha }+{{\varvec{v}}}_{{\varvec{j}}\boldsymbol{ }}+\boldsymbol{ }{{\varvec{u}}}_{{\varvec{j}}({\varvec{r}})}$$

7 $${{\varvec{u}}}_{{\varvec{j}}({\varvec{r}})\boldsymbol{ }}\sim {\varvec{C}}{\varvec{A}}{\varvec{R}}({\varvec{\tau}},{\varvec{W}})$$

8 $${\sum }_{{\varvec{r}}}{\sum }_{{\varvec{j}}\in {\varvec{r}}}{{\varvec{u}}}_{{\varvec{j}}({\varvec{r}})}=0.$$

By setting the option adjust.for.con.comp = FALSE, INLA interprets this as a sum-to-zero constraint for the union of the subgraphs. This is specified in INLA as:

formula = y ~ 1 + f(struct, model='besag', graph=W.graph, adjust.for.con.comp = FALSE, scale.model = TRUE) + f(unstruct, model ='iid')

In this formulation, “struct” and “unstruct” correspond to a vector (1, …, N) with a number of areas N, and “W.graph” is the neighbourhood structure. The specification scale.model = TRUE defines a scaled version of the random effects such that the variance parameter can be interpreted as a marginal variance.

### Model II: sum-to-zero constraints for each region

When specifying the graph for the ICAR prior in INLA, INLA interprets this as a sum-to-zero constraint for each subgraph by default, imposing a separate sum-to-zero constraint of the random effects for each region ${\varvec{r}}$.

9 $${\varvec{l}}{\varvec{o}}{\varvec{g}}{\varvec{i}}{\varvec{t}}({{\varvec{p}}}_{{\varvec{i}}{\varvec{j}}({\varvec{r}})})=\boldsymbol{\alpha }+{{\varvec{v}}}_{{\varvec{j}}\boldsymbol{ }}+\boldsymbol{ }{{\varvec{u}}}_{{\varvec{j}}({\varvec{r}})}$$

10 $${{\varvec{u}}}_{{\varvec{j}}({\varvec{r}})\boldsymbol{ }}\sim {\varvec{C}}{\varvec{A}}{\varvec{R}}({\varvec{\tau}},{\varvec{W}})$$

11 $$\forall {\varvec{r}}:\boldsymbol{ }{\sum }_{{\varvec{j}}\in {\varvec{r}}}{{\varvec{u}}}_{{\varvec{j}}({\varvec{r}})}=0$$

This model assumes a common intercept for all disconnected regions, so the overall risk in the separate regions is the same. The spatial random effects ${{\varvec{u}}}_{{\varvec{j}}({\varvec{r}})}$ in this case need to be interpreted as the area-specific deviation from the overall risk, which varies around zero in each disconnected region.

This default setting is equivalent to setting the option adjust.for.con.comp = TRUE in INLA, such that a separate sum-to-zero constraint of the random effects is applied for each region.

formula = y ~ 1 + f(struct, model = 'besag', graph = W.graph, adjust.for.con.comp = TRUE, scale.model = TRUE) + f(unstruct, model ='iid')

In this case, scaling is done with respect to each subgraph.

### Model III: sum-to-zero constraint and intercept for each region

A more flexible model assigns one intercept to each region in addition to using a sum-to-zero constraint for each connected region. By adding an intercept for each region, we infer that the baseline prevalence is different in the disconnected regions.

12 $${\varvec{l}}{\varvec{o}}{\varvec{g}}{\varvec{i}}{\varvec{t}}({{\varvec{p}}}_{{\varvec{i}}{\varvec{j}}({\varvec{r}})})={\boldsymbol{\alpha }}_{{\varvec{r}}}+{{\varvec{v}}}_{{\varvec{j}}\boldsymbol{ }}+\boldsymbol{ }{{\varvec{u}}}_{{\varvec{j}}({\varvec{r}})}$$

13 $${{\varvec{u}}}_{{\varvec{j}}({\varvec{r}})\boldsymbol{ }}\sim {\varvec{C}}{\varvec{A}}{\varvec{R}}({\varvec{\tau}},{\varvec{W}})$$

14 $$\forall {\varvec{r}}:\boldsymbol{ }{\sum }_{{\varvec{j}}\in {\varvec{r}}}{{\varvec{u}}}_{{\varvec{j}}({\varvec{r}})}=0$$

The spatial random effects ${{\varvec{u}}}_{{\varvec{j}}({\varvec{r}})}$ can be interpreted as the area-specific deviation from the region-specific risk in this case. The intercepts for the disconnected regions need to be explicitly specified in INLA:

formula = y ~ -1 + region + f(struct, model='besag', graph=W.graph, adjust.for.con.comp = TRUE, scale.model = TRUE) + f(unstruct, model ='iid')

### Model IV: split random effects model

Models 1–3 assume one random effects distribution for all areas in the region, with the random effects variance corresponding to the variation of the random effects over all areas in all the disconnected regions. As the disconnected regions can be quite different in terms of demographics, socioeconomics, infrastructure, and development, we can also assume that the variation in these regions is different. A separate analysis could be conducted for the two regions, but this would be restrictive in the model comparison (e.g., the use of DIC or WAIC for model comparison would not be possible in this case). Therefore, a split random effect is proposed so that use of DIC and WAIC are still possible to assess the model fit.

15 $${\varvec{l}}{\varvec{o}}{\varvec{g}}{\varvec{i}}{\varvec{t}}\left({{\varvec{p}}}_{{\varvec{i}}{\varvec{j}}\left({\varvec{r}}\right)}\right)={\boldsymbol{\alpha }}_{{\varvec{r}}}+{{\varvec{v}}}_{{\varvec{j}}\boldsymbol{ }}+\boldsymbol{ }{{\varvec{u}}}_{{\varvec{j}}\left({\varvec{r}}\right)}$$

16 $${\forall {\varvec{r}}: {\varvec{u}}}_{{\varvec{j}}({\varvec{r}})\boldsymbol{ }}\sim {\varvec{C}}{\varvec{A}}{\varvec{R}}\left({{\varvec{\tau}}}_{{\varvec{r}}},{{\varvec{W}}}_{{\varvec{r}}}\right)$$

17 $$\forall {\varvec{r}}:\boldsymbol{ }{\sum }_{{\varvec{j}}\in {\varvec{r}}}{{\varvec{u}}}_{{\varvec{j}}({\varvec{r}})}=0$$

This model assumes separate ICAR random effects for the disconnected regions, so each subgraph has its own spatial variance. These random effects are defined in the subgraph ${{\varvec{W}}}_{{\varvec{r}}}$ for a sub-region ${\varvec{r}}$ and have a separate spatial precision. This can be specified in INLA as follows:

formula = y ~ -1 + region + f(struct1, model = 'besag', graph = W.graph1, scale.model = TRUE) + f(unstruct1, model = 'iid') + f(struct2, model = 'besag', graph = W.graph2, scale.model = TRUE) + f(unstruct2, model = 'iid')

In this specification, “struct1” and “unstruct1” correspond to a vector (1_1_, …, N_1_), where N_1_ is the number of areas in region 1, and “W.graph1” is the neighbourhood structure amongst these areas. “struct2”, “unstruct2”, and “W.graph2” correspond to similar properties for region 2.

## Results

**Fig. 1:**
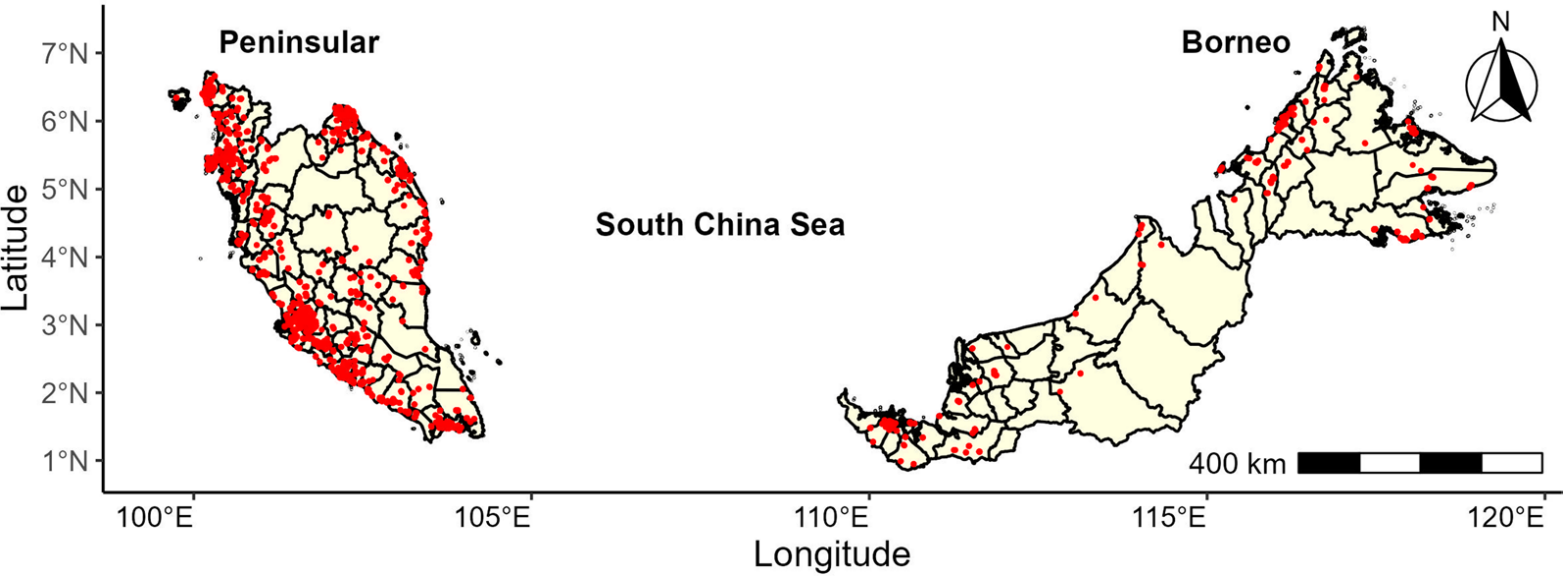
Geographical distribution of participating children across districts in Malaysia, National Health and Morbidity Survey 2015

A total of 6,812 children aged 5 to 17 years participated in NHMS 2015. For BMI, 5% of the data were missing, while 2% of the geolocation information were missing or invalid. Figure 1 shows the geographical distribution of participating children with complete BMI and geolocation information (n = 6301). The samples range from 0 to 363 per district (Median = 28). There were 85 districts (59%) that had sample sizes less than 50, and 18 districts had no samples. This indicates high variability in the sample sizes between districts. The national prevalence of overweight (including obesity) was 23.8% (95%CI 22.2, 25.4), the prevalence for boys was 24.5% (95%CI 22.3, 26.9), and the prevalence for girls was 23.0% (95%CI 21.1, 24.9).

### Geographical variation in prevalence of overweight

The main focus of this study was to explore the geographical variation in the prevalence of overweight in children aged 5 to 17 years and to identify districts with high prevalence. The direct estimate of district-level prevalence of overweight in sampled areas was 23.4% (SE 1.25). The lowest was in the district Lahad Datu at 1.6% (SE 1.6), and the highest was in the district Sarikei at 82.7% (SE 16.8) (see Additional file 1).

**Fig. 2:**
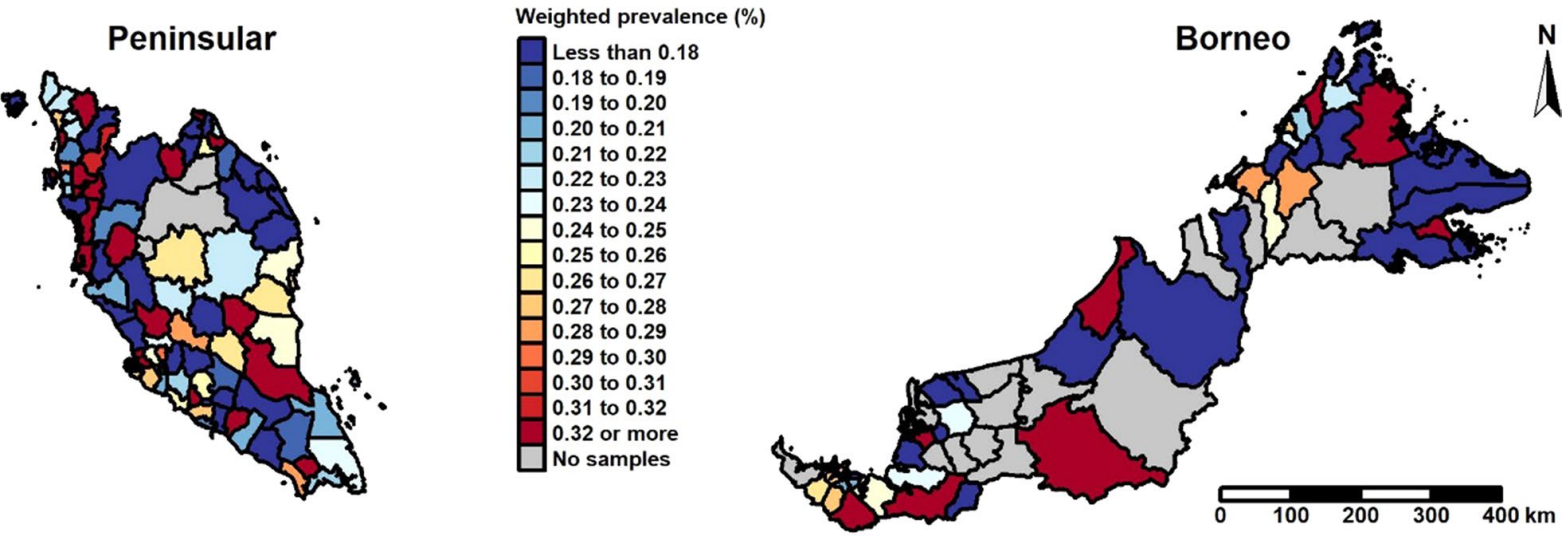
District-level weighted overweight prevalence of children aged 5–17 years, NHMS 2015, Malaysia

Figure 2 illustrates the design-based weighted prevalence of overweight per district (see Additional file 2 for the uncertainty of the prevalence estimates). Substantial heterogeneities can be observed across districts. Districts with high overweight prevalence can be seen next to districts with low overweight prevalence. Areas indicated in grey show districts where no estimates could be obtained as there were no samples in these areas.

Table 2 shows the posterior summary statistics of the different models considered in the analysis. Model IVa (with a common intercept specification and split random effects) had the lowest DIC and WAIC values. The posterior summary statistics of the model with split random effects and separate intercepts are also shown (Model IVb). Upon comparison, Models Va and Vb showed the summary statistics of the models when separate analysis was conducted for the Borneo and Peninsular regions. These analyses showed that when specifying split random effects and separate intercepts (Model IVb), it is the same as running two separate models (Models Va and Vb) but with the advantage of one DIC/ WAIC value being obtained to assess the model fit and the possibility of testing for a common effect (such as a common intercept).

**Table 2:** Posterior summary statistics of the different models

	Model I	Model II	Model III	Model IVa	Model IVb	Model Va	Model Vb
	Mean (95% Credible interval)	Mean (95% Credible interval)	Mean (95% Credible interval)	Mean (95% Credible interval)	Mean (95% Credible interval)	Mean (95% Credible interval)	Mean (95% Credible interval)
Intercept							
Malaysia	− 1.192 (− 1.297, − 1.085)	− 1.174 (− 1.261, − 1.087)		− 1.173 (− 1.262, − 1.084)			
Borneo			− 1.208 (− 1.421, − 0.996)		− 1.205 (− 1.421, − 0.986)	− 1.205 (− 1.427, − 0.983)	
Peninsular			− 1.167 (− 1.263, − 1.070)		− 1.166 (− 1.265, − 1.067)		− 1.167 (− 1.261, − 1.072)
Variance							
Malaysia							
Structured RE^a^	0.109 (0.001, 0.780)	0.020 (0.002, 0.079)	0.019 (0.002, 0.074)				
Unstructured RE^a^	0.082 (0.033, 0.160)	0.066 (0.021, 0.147)	0.069 (0.024, 0.148)				
Borneo							
Structured RE^a^				0.033 (0.020, 0.040)	0.023 (0.018, 0.025)	0.071 (0.006, 0.363)	
Unstructured RE^a^				0.051 (0.038, 0.099)	0.051 (0.036, 0.077)	0.060 (0.003, 0.228)	
Peninsular							
Structured RE^a^				0.010 (0.002, 0.029)	0.006 (0.002, 0.009)		0.018 (0.002, 0.081)
Unstructured RE^a^				0.056 (0.017, 0.119)	0.064 (0.022, 0.138)		0.059 (0.017, 0.135)
DIC^b^	206.4	203.8	205.3	203.2	204.5	85.7	121.2
WAIC^c^	208.1	206.3	207.9	205.4	207.1	87.2	123.4

Model I: A single sum-to-zero constraint (adjust.for.con.comp = FALSE) and a common intercept

Model II: Sum-to-zero constraints for each region (adjust.for.con.comp = TRUE) and a common intercept

Model III: Sum-to-zero constraint (adjust.for.con.comp = TRUE) and intercept for each region

Model IVa: Split random effects and a common intercept

Model IVb: Split random effects and intercept for each region

Model Va: Separate analysis for Borneo region only

Model Vb: Separate analysis for Peninsular region only

^a^ RE: random effects

^b^ DIC: Deviance information criterion [24]

^c^ WAIC: Watanabe information criterion [25]

Using Model IVa, we introduced each district-level covariate separately (as shown in Table 1) and tested each covariate for an interaction effect with the Region variable. Table 3 shows the best-fit model with the lowest DIC and WAIC after adjusting for district-level covariates. No significant associations were observed between other covariates with overweight prevalence except for the proportion of Bumiputera ethnicity. There was a significant negative association between the proportion of Bumiputera ethnicity and prevalence of overweight in the district: the higher the proportion of Bumiputera ethnicity in the district, the lower the prevalence of childhood overweight. The same effect applies to both regions, but the amount of spatial variation is different between them.

**Table 3:** Posterior summary statistics of the best-fit adjusted model

District-level covariate	Mean	95% Credible interval	
		Lower	Upper
Intercept	− 1.204	− 1.294	− 1.113
% Bumiputera ethnicity^a^	− 0.101	− 0.191	− 0.010
Variance			
Borneo			
Structured RE^b^	0.035	0.009	0.101
Unstructured RE^b^	0.052	0.008	0.164
Peninsular			
Structured RE^b^	0.014	0.002	0.057
Unstructured RE^b^	0.044	0.010	0.110
DIC^c^	203.7		
WAIC^d^	206.2		

^a^Proportion of Bumiputera ethnicity at the district level. Bumiputera ethnicity comprises of Malay, Orang Asli, Sabah Bumiputera and Sarawak Bumiputera

^b^RE: Random effects

^c^DIC: Deviance information criterion [24]

^d^WAIC: Watanabe information criterion [25]

**Fig. 3:**
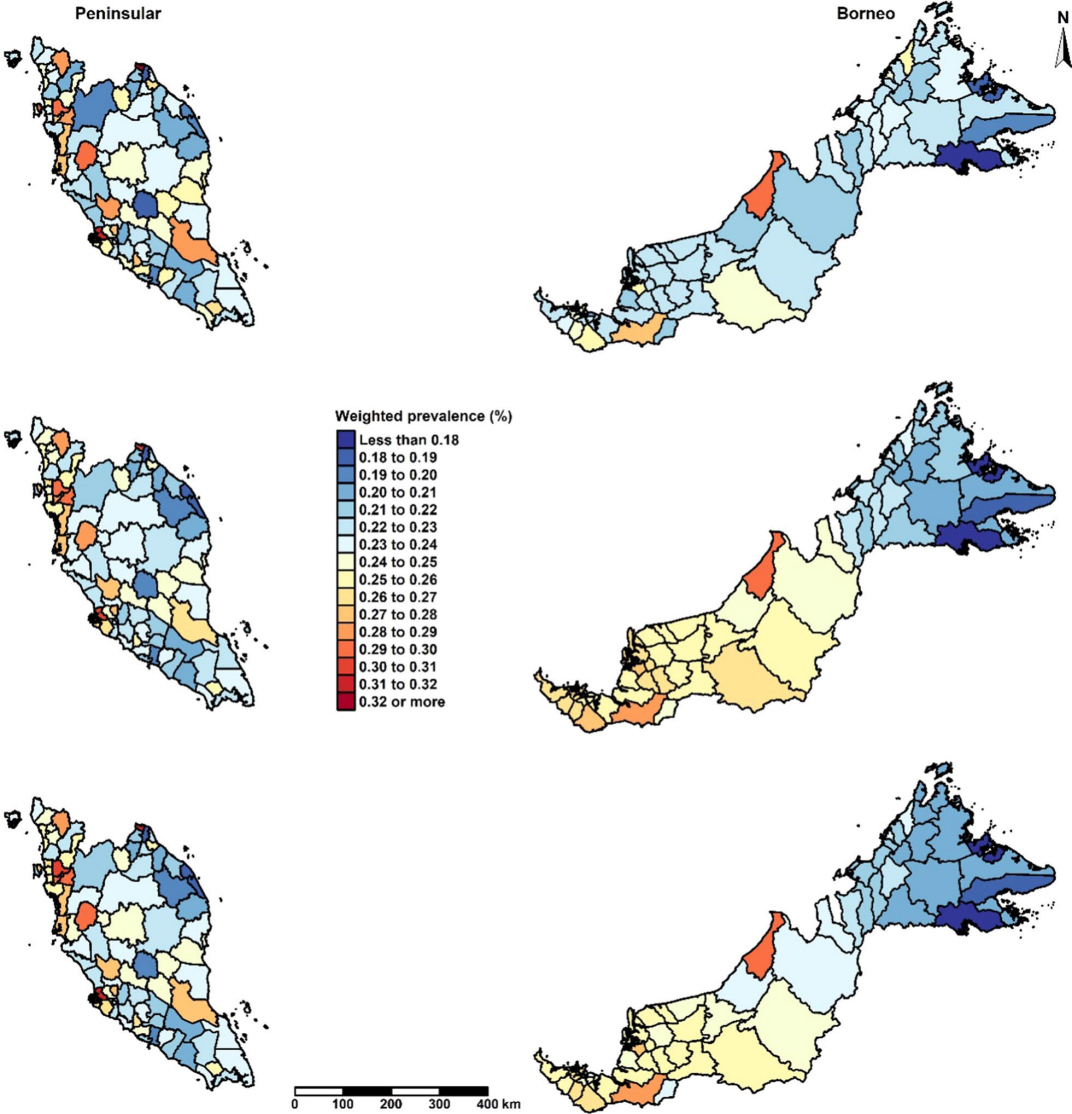
District-level predicted prevalence of overweight in children aged 5–17 years old, NHMS 2015, Malaysia. The top map corresponds to Model I, a single sum-to-zero constraint. The middle map corresponds to Model II, sum-to-zero constraints for each region and a common intercept. The bottom map corresponds to Model III, sum-to-zero constraint and intercept for each region

Figure 3 illustrates the effects of spatial smoothing on the prevalence estimates (see Additional file 2 for the uncertainty of the prevalence estimates). The top map corresponds to Model I with a single sum-to-zero constraint for both regions. The middle and bottom maps correspond to Model II (sum-to-zero constraints for each region) and Model III (sum-to-zero constraint and intercept for each region), respectively. These district-level maps reveal a spatial pattern in the prevalence of overweight. No obvious difference can be seen in prevalence estimates in the Peninsular region across the three models. Districts located in the eastern, northern, and southern part of the Peninsular region have a lower prevalence of overweight than districts located in the northwest and central west of this region. An increasing trend in the prevalence can be seen when moving from the east to the west part of the Peninsular region.

Districts in Borneo showed some variations in the different model specifications. A more homogeneous pattern in overweight estimates can be observed across districts in Borneo in the top map of Fig. 3 (with a single sum-to-zero constraint) than in the middle and bottom maps (with sum-to-zero constraints for each region). An increasing trend in the prevalence was observed when moving from the east to the west part of Borneo. A more distinct pattern occurred when a similar baseline mean was specified in these two regions, as evident in the middle map in Fig. 3. These variations in the different model specifications seen in Borneo can be attributed to the fewer data available in this region: 15 of the 57 districts in Borneo had no observations, so the outcome was more dependent on model assumptions. Thus, the choice of the model can seriously impact the result.

**Fig. 4:**
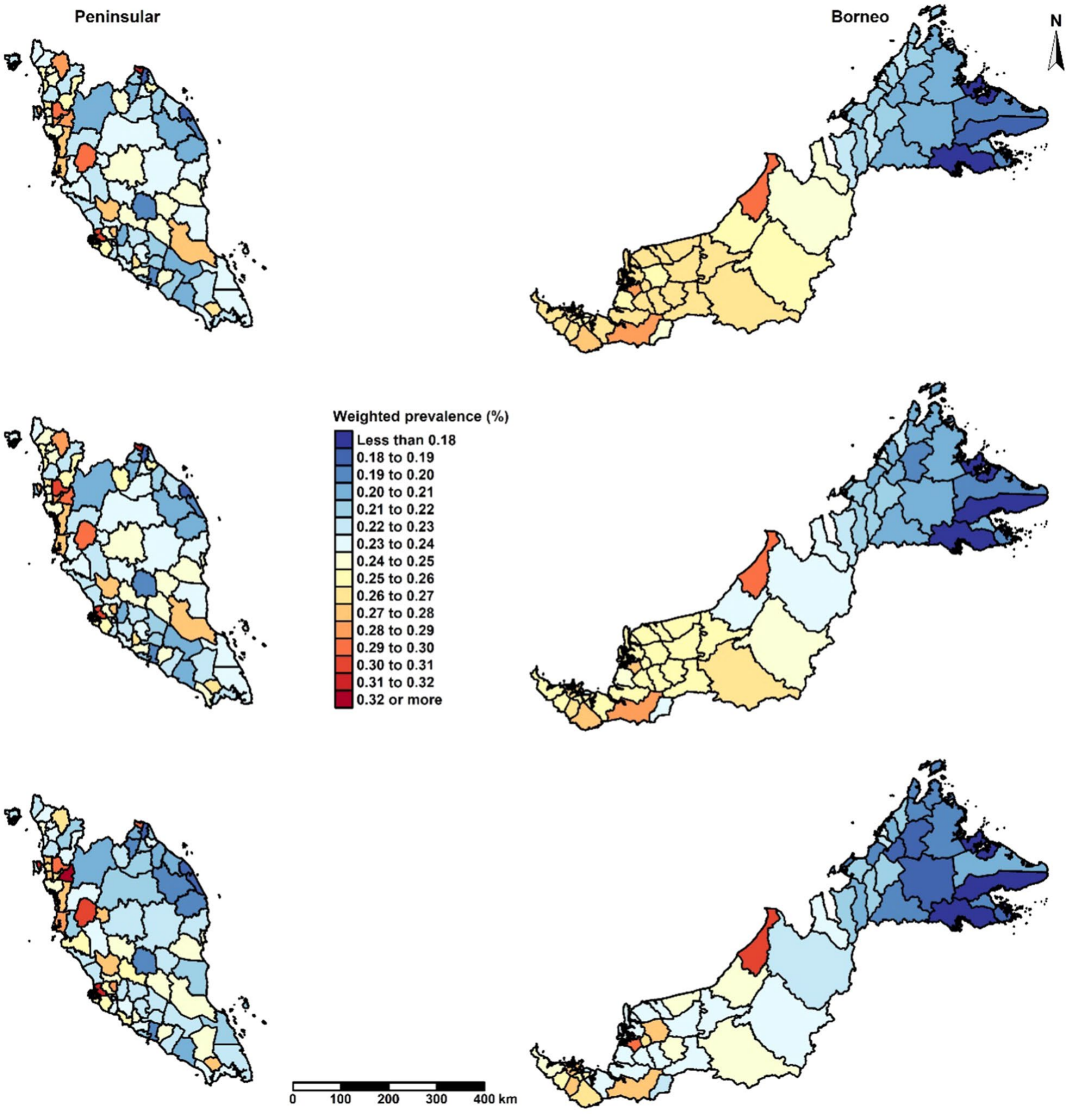
District-level predicted prevalence of overweight in children aged 5–17 years old, NHMS 2015, Malaysia. The top map corresponds to Model IVa, split random effects (RE) with a common intercept. The middle map corresponds to Model IVb, split RE with separate intercepts. The bottom map corresponds to Model IVa adjusted for district-level covariate (Proportion of Bumiputera ethnicity)

Figure 4 illustrates the effect of split random effects specification and the addition of covariates on the smoothed prevalence estimates (see Additional file 2 for the uncertainty of the prevalence estimates). The top and middle maps correspond to Models IVa and IVb, respectively, as presented in Table 2. The pattern in the top map of Fig. 4 (split random effects with a common intercept) was similar to the middle map in Fig. 3 (sum-to-zero constraints for each region). The pattern in the middle map in Fig. 4 was similar to the bottom map in Fig. 3 (sum-to-zero constraint and intercept for each region). A reduction in variance of the prevalence estimates can be seen in the bottom map in Fig. 4 when adjusting the model with the proportion of Bumiputera ethnicity in the district.

## Discussion

This paper provides the first overview of different spatial model types when dealing with a study area that is split into separate disconnected regions. This has not yet been discussed widely in the literature. The presented software implementation in R-INLA, a widely used R package for spatial disease mapping, can help to practically implement the different model types. While methods are illustrated in the context of a spatial health survey with complex sampling designs, the different model types are applicable in a broader context whenever a study region is split into disconnected regions.

In this study, we found that the model with split random effects and a common intercept (a similar baseline mean) in the two regions (Model IVa) was the model with the best fit for describing the prevalence of childhood overweight across districts. Based on this selected model, a common level of childhood overweight prevalence is apparent in the two regions, but with different spatial variations between them. This highlights the importance of exploring different types of spatial models when mapping disease prevalence to find the best model, especially when dealing with disconnected regions.

We estimated that around 24% of Malaysian children aged 5–17 years had overweight in 2015 (approximately 1.4 million children). The NHMS provided estimates at the national and state level, but these estimates could mask variations at the district level. Our study is the first to report estimates of overweight among children in this age group at the district level, which could help in monitoring and addressing the overweight epidemic at the subnational level in Malaysia.

The results showed that there was a two-fold variation in the prevalence of overweight among children between districts in Malaysia (17–34%). Our study also revealed that there was a spatial pattern in overweight occurrences across districts with increasing trends observed when moving from east to west in the Peninsular and Borneo regions. These spatial disparities can be attributed to many factors, including compositional factors such as differences in demographic and socioeconomic backgrounds of the population living in the area. Possible contextual factors include differences in the development, infrastructure, and built environments between the areas. These factors warrant further assessment.

When the selected model was further adjusted with district-level covariates, our results revealed a significant negative association between the proportion of Bumiputera ethnicity in the districts and the prevalence of childhood overweight. The Bumiputera ethnicity comprises the Malay, Orang Asli, Sabah Bumiputera, and Sarawak Bumiputera ethnicities and constitutes the main ethnic group in all states except for Penang state, which is located in the northwest of the Peninsular region, and the capital city Kuala Lumpur, which is located in the central west of the Peninsular region [28]. No previous local studies have reported associations of district-level determinants and overweight prevalence in children for comparison.

To our knowledge, this is the first study to use data from a large nationally representative survey to obtain subnational prevalence estimates of overweight among Malaysian children. Using Bayesian hierarchical modelling enabled us to predict prevalence in unsampled districts. We also illustrated techniques for the specification of graphs for disconnected regions. In addition, complex survey design was considered in all analyses to reduce bias from non-response and non-random sampling. Regarding limitations, we have specified neighbourhood structure using the most widely used definition, i.e., two areas are neighbours if they share a common border (contiguity). However, there are other approaches, such as the distance-based method, which measures the distance between two points or centroids, that are worth further investigation in future research. In addition, the association observed at the aggregate level in this ecological study cannot be inferred to the individual level. Thus, both district- and individual-level covariates should be included in the mapping of overweight prevalence in future research.

## Conclusion

The findings from this study have several implications. First, we described different model specifications for disease mapping of disconnected regions using R-INLA. From this description, we demonstrated techniques that can be utilized to produce reliable subnational estimates of disease prevalence for disconnected regions using survey data. This is much needed information for local stakeholders for prioritized intervention and optimal planning. Second, using mapping of overweight prevalence among children in Malaysia as an example, we showed that overweight occurrences among children in Malaysia followed a spatial pattern with an east–west gradient trend. We also identified districts with high prevalence of overweight. This could help policy makers in making informed decisions to enhance public health interventions in high-risk districts to curb the overweight epidemic in Malaysia.

## Supplementary Information

**Additional file 1: **Posterior summary statistics of design-based prevalence estimates and model-based prevalence estimates of overweight, NHMS 2015, Malaysia.

**Additional file 2: Figure S1.** The uncertainty in the prevalence estimates of overweight shown in Fig. 2. **Figure S2.** The uncertainty in the respective estimated prevalence of overweight shown in Fig. 3. The top map corresponds to Model I, a single sum-to-zero constraint. The middle map corresponds to Model II, sum-to-zero constraints for each region and a common intercept. The bottom map corresponds to Model III, sum-to-zero constraint and intercept for each region. **Figure S3.** The uncertainty in the respective estimated prevalence of overweight shown in Fig. 4. The top map corresponds to Model IVa, split random effects with a common intercept. The middle map corresponds to Model IVb, split RE with separate intercepts. The bottom map corresponds to Model IVa adjusted for district-level covariate.

## Data Availability

The data that support the findings of this study are not publicly available due to privacy or ethical restrictions. We will provide a simulated data which reflects the characteristics of the original data used in this study and the code used for the simulation.

## Abbreviations

BMI: Body mass index
CI: Confidence interval
DIC: Deviance information criterion
ICAR: Intrinsic conditional autoregressive
INLA: Integrated Nested Laplace Approximation
IOTF: International Obesity Taskforce
NHMS: National Health and Morbidity Survey
RE: Random effects
SE: Standard error
WAIC: Watanabe information criterion
