# Supplementary material for: A practical illustration of spatial smoothing methods for disconnected regions with INLA: spatial survey on overweight and obesity in Malaysia
Source: Int J Health Geogr. 2023 Jun 21;22:14. doi: 10.1186/s12942-023-00336-5 (PMC10286432; doi:10.1186/s12942-023-00336-5)
Supplement: Supplementary file 1 — Additional file 1: Posterior summary statistics of design-based prevalence estimates and model-based prevalence estimates of overweight, NHMS 2015, Malaysia. [file 12942_2023_336_MOESM1_ESM.pdf]

Additional File 1

A practical illustration of spatial smoothing methods for disconnected regions with INLA: spatial survey on overweight and obesity in Malaysia

Posterior summary statistics of design-based (weighted, unsmoothed) prevalence estimates and model-based (weighted, smoothed) prevalence estimates of overweight (including obesity), NHMS 2015, Malaysia.

Districts	Regions	p.hat1	se.p.hat1	p.hat2	logit.p1	logit.p2	logit.v1	logit.v2
Batu Pahat	Peninsular	0.164	0.042	0.208	-1.631	-1.335	0.095	0.038
Johor Bahru	Peninsular	0.220	0.031	0.233	-1.266	-1.194	0.032	0.022
Kluang	Peninsular	0.186	0.107	0.241	-1.477	-1.149	0.503	0.053
Kota Tinggi	Peninsular	0.234	0.063	0.220	-1.186	-1.264	0.124	0.041
Kulai	Peninsular	0.342	0.087	0.271	-0.656	-0.991	0.150	0.046
Tangkak	Peninsular	0.495	0.251	0.248	-0.021	-1.108	1.009	0.058
Mersing	Peninsular	0.202	0.178	0.214	-1.375	-1.299	1.217	0.058
Muar	Peninsular	0.206	0.053	0.229	-1.352	-1.215	0.104	0.038
Pontian	Peninsular	0.288	0.121	0.236	-0.906	-1.174	0.348	0.052
Segamat	Peninsular	0.050	0.035	0.221	-2.941	-1.259	0.545	0.055
Baling	Peninsular	0.311	0.124	0.238	-0.794	-1.162	0.334	0.051
Bandar Baharu	Peninsular	0.412	0.110	0.268	-0.356	-1.007	0.205	0.051
Kota Setar	Peninsular	0.275	0.094	0.249	-0.969	-1.102	0.220	0.048
Kuala Muda	Peninsular	0.198	0.040	0.229	-1.401	-1.216	0.065	0.032
Kubang Pasu	Peninsular	0.222	0.088	0.232	-1.251	-1.197	0.257	0.051
Kulim	Peninsular	0.384	0.069	0.293	-0.475	-0.881	0.085	0.039
Langkawi	Peninsular	0.080	0.077	0.220	-2.445	-1.265	1.097	0.065
Padang Terap	Peninsular	0.374	0.081	0.265	-0.514	-1.020	0.121	0.047
Pendang	Peninsular	0.222	0.086	0.231	-1.255	-1.201	0.250	0.049
Pokok Sena	Peninsular	0.229	0.121	0.231	-1.212	-1.203	0.466	0.057
Sik	Peninsular	0.133	0.059	0.212	-1.877	-1.313	0.260	0.052
Yan	Peninsular	0.323	0.143	0.238	-0.742	-1.165	0.430	0.057
Bachok	Peninsular	0.222	0.052	0.216	-1.253	-1.287	0.091	0.038
Gua Musang	Peninsular			0.216		-1.289		0.057
Jeli	Peninsular	0.332	0.120	0.226	-0.698	-1.230	0.294	0.051
Kota Bharu	Peninsular	0.166	0.024	0.187	-1.615	-1.471	0.030	0.021
Kuala Krai	Peninsular			0.214		-1.300		0.058
Machang	Peninsular	0.251	0.127	0.217	-1.093	-1.285	0.458	0.052
Pasir Mas	Peninsular	0.174	0.061	0.207	-1.557	-1.344	0.178	0.045
Pasir Puteh	Peninsular	0.405	0.126	0.235	-0.386	-1.180	0.275	0.055
Tanah Merah	Peninsular	0.164	0.043	0.198	-1.626	-1.398	0.097	0.037
Tumpat	Peninsular	0.470	0.082	0.290	-0.120	-0.895	0.108	0.057
Kuala Lumpur	Peninsular	0.298	0.038	0.284	-0.855	-0.924	0.032	0.021
Labuan	Borneo	0.141	0.081	0.202	-1.806	-1.377	0.450	0.081
Alor Gajah	Peninsular	0.273	0.067	0.236	-0.979	-1.176	0.115	0.039
Jasin	Peninsular	0.092	0.041	0.195	-2.292	-1.418	0.238	0.052
Melaka Tengah	Peninsular	0.199	0.039	0.220	-1.394	-1.266	0.059	0.032
Jelebu	Peninsular	0.137	0.099	0.228	-1.840	-1.219	0.696	0.052
Jempol	Peninsular	0.189	0.052	0.221	-1.458	-1.262	0.117	0.037
Kuala Pilah	Peninsular	0.253	0.112	0.226	-1.082	-1.229	0.352	0.049
Port Dickson	Peninsular	0.254	0.093	0.254	-1.078	-1.076	0.241	0.047
Rembau	Peninsular	0.361	0.090	0.245	-0.573	-1.123	0.152	0.047
Seremban	Peninsular	0.216	0.035	0.233	-1.288	-1.191	0.043	0.024
Tampin	Peninsular	0.170	0.065	0.227	-1.587	-1.226	0.212	0.045
Bentong	Peninsular	0.290	0.095	0.257	-0.896	-1.064	0.212	0.043

Bera	Peninsular	0.269	0.076	0.244	-1.001	-1.128	0.148	0.039
Cameron Highlands	Peninsular			0.278		-0.955		0.067
Jerantut	Peninsular	0.225	0.077	0.223	-1.239	-1.249	0.198	0.041
Kuantan	Peninsular	0.267	0.051	0.244	-1.010	-1.132	0.069	0.031
Lipis	Peninsular	0.268	0.113	0.227	-1.007	-1.226	0.335	0.048
Maran	Peninsular	0.323	0.112	0.226	-0.739	-1.230	0.260	0.049
Pekan	Peninsular	0.240	0.067	0.219	-1.151	-1.270	0.135	0.042
Raub	Peninsular	0.225	0.103	0.243	-1.238	-1.135	0.349	0.047
Rompin	Peninsular	0.515	0.133	0.243	0.061	-1.137	0.285	0.058
Temerloh	Peninsular	0.124	0.039	0.199	-1.953	-1.394	0.127	0.043
Batang Padang	Peninsular	0.159	0.065	0.232	-1.663	-1.197	0.233	0.045
Hilir Perak	Peninsular	0.208	0.078	0.253	-1.335	-1.084	0.226	0.048
Hulu Perak	Peninsular	0.132	0.044	0.205	-1.886	-1.356	0.150	0.044
Kampar	Peninsular	0.714	0.147	0.327	0.915	-0.723	0.516	0.066
Kerian	Peninsular	0.162	0.106	0.244	-1.640	-1.133	0.612	0.057
Kinta	Peninsular	0.343	0.051	0.306	-0.652	-0.819	0.050	0.029
Kuala Kangsar	Peninsular	0.198	0.054	0.237	-1.399	-1.167	0.117	0.038
Larut Dan Matang	Peninsular	0.375	0.105	0.278	-0.509	-0.953	0.200	0.046
Manjung	Peninsular	0.363	0.087	0.285	-0.561	-0.921	0.142	0.043
Perak Tengah	Peninsular	0.147	0.136	0.213	-1.761	-1.306	1.186	0.058
Perlis	Peninsular	0.228	0.024	0.229	-1.222	-1.212	0.018	0.014
Barat Daya	Peninsular	0.178	0.073	0.235	-1.532	-1.183	0.249	0.050
Seberang Perai Selatan	Peninsular	0.214	0.077	0.275	-1.300	-0.969	0.208	0.054
Seberang Perai Tengah	Peninsular	0.201	0.098	0.269	-1.381	-1.000	0.373	0.059
Seberang Perai Utara	Peninsular	0.292	0.072	0.273	-0.885	-0.980	0.120	0.041
Timur Laut	Peninsular	0.343	0.083	0.312	-0.651	-0.791	0.137	0.051
Putrajaya	Peninsular	0.233	0.035	0.223	-1.190	-1.247	0.038	0.026
Beaufort	Borneo	0.281	0.146	0.210	-0.939	-1.323	0.523	0.072
Beluran	Borneo	0.500	0.354	0.195	0.000	-1.417	2.006	0.089
Keningau	Borneo	0.280	0.096	0.215	-0.943	-1.298	0.225	0.060
Kinabatangan	Borneo	0.000	0.000	0.201		-1.379		0.098
Kota Belud	Borneo	0.546	0.191	0.215	0.186	-1.293	0.592	0.095
Kota Kinabalu	Borneo	0.273	0.060	0.238	-0.977	-1.163	0.092	0.044
Kota Marudu	Borneo	0.224	0.088	0.198	-1.245	-1.398	0.255	0.064
Kuala Penyu	Borneo			0.198		-1.396		0.097
Kudat	Borneo	0.000	0.000	0.198		-1.399		0.128
Kunak	Borneo	0.373	0.287	0.198	-0.518	-1.397	1.506	0.100
Lahad Datu	Borneo	0.016	0.016	0.175	-4.139	-1.549	1.033	0.103
Nabawan	Borneo			0.193		-1.432		0.087
Papar	Borneo	0.000	0.000	0.199		-1.390		0.083
Penampang	Borneo	0.232	0.079	0.224	-1.199	-1.243	0.196	0.053
Pitas	Borneo	0.000	0.000	0.191		-1.446		0.107
Putatan	Borneo	0.120	0.053	0.187	-1.988	-1.472	0.253	0.068
Ranau	Borneo	0.080	0.077	0.186	-2.437	-1.473	1.088	0.079
Sandakan	Borneo	0.069	0.034	0.178	-2.600	-1.530	0.287	0.102
Semporna	Borneo	0.000	0.000	0.184		-1.490		0.116
Sipitang	Borneo			0.208		-1.334		0.084
Tambunan	Borneo	0.141	0.093	0.188	-1.805	-1.460	0.591	0.071
Tawau	Borneo	0.146	0.026	0.173	-1.765	-1.566	0.044	0.034
Tenom	Borneo	0.245	0.069	0.213	-1.127	-1.305	0.138	0.048
Tongod	Borneo			0.190		-1.451		0.086
Tuaran	Borneo	0.212	0.069	0.202	-1.312	-1.372	0.172	0.051
Asajaya	Borneo	0.113	0.076	0.223	-2.062	-1.247	0.573	0.096
Bau	Borneo	0.260	0.114	0.253	-1.045	-1.080	0.349	0.085
Belaga	Borneo			0.234		-1.188		0.094
Betong	Borneo	0.237	0.105	0.238	-1.172	-1.164	0.339	0.073
Bintulu	Borneo	0.093	0.088	0.241	-2.278	-1.147	1.098	0.087
Dalat	Borneo	0.000	0.000	0.238		-1.162		0.096
Daro	Borneo			0.236		-1.177		0.099
Julau	Borneo			0.236		-1.175		0.092
Kanowit	Borneo			0.243		-1.138		0.093
Kapit	Borneo	0.428	0.206	0.249	-0.290	-1.103	0.706	0.085
Kuching	Borneo	0.278	0.059	0.277	-0.954	-0.961	0.085	0.044

Lawas	Borneo	0.093	0.090	0.213	-2.273	-1.306	1.139	0.085
Limbang	Borneo			0.231		-1.204		0.102
Lubok Antu	Borneo	0.137	0.080	0.226	-1.841	-1.232	0.458	0.084
Lundu	Borneo			0.245		-1.125		0.120
Marudi	Borneo	0.171	0.105	0.226	-1.576	-1.233	0.549	0.076
Matu	Borneo	0.000	0.000	0.235		-1.180		0.102
Maradong	Borneo	0.000	0.000	0.269		-1.002		0.091
Miri	Borneo	0.489	0.107	0.307	-0.046	-0.816	0.184	0.086
Mukah	Borneo			0.241		-1.150		0.098
Pakan	Borneo			0.236		-1.172		0.095
Samarahan	Borneo	0.207	0.140	0.250	-1.343	-1.097	0.728	0.084
Saratok	Borneo	0.168	0.110	0.234	-1.603	-1.187	0.617	0.086
Sarikei	Borneo	0.827	0.168	0.297	1.567	-0.861	1.379	0.098
Selangau	Borneo			0.236		-1.175		0.092
Serian	Borneo	0.371	0.129	0.260	-0.528	-1.045	0.306	0.078
Sibu	Borneo	0.235	0.079	0.279	-1.180	-0.950	0.195	0.059
Simunjan	Borneo	0.240	0.151	0.237	-1.152	-1.172	0.682	0.082
Song	Borneo			0.237		-1.168		0.089
Sri Aman	Borneo	0.600	0.159	0.276	0.404	-0.965	0.439	0.089
Tatau	Borneo			0.237		-1.169		0.090
Gombak	Peninsular	0.235	0.043	0.241	-1.182	-1.146	0.057	0.027
Hulu Langat	Peninsular	0.176	0.040	0.223	-1.544	-1.249	0.075	0.034
Hulu Selangor	Peninsular	0.356	0.072	0.271	-0.591	-0.988	0.098	0.038
Klang	Peninsular	0.354	0.044	0.312	-0.602	-0.792	0.036	0.025
Kuala Langat	Peninsular	0.278	0.052	0.258	-0.954	-1.054	0.068	0.031
Kuala Selangor	Peninsular	0.159	0.061	0.221	-1.665	-1.262	0.211	0.045
Petaling	Peninsular	0.244	0.033	0.251	-1.130	-1.094	0.033	0.020
Sabak Bernam	Peninsular	0.166	0.065	0.221	-1.614	-1.260	0.217	0.046
Sepang	Peninsular	0.208	0.056	0.230	-1.335	-1.206	0.114	0.036
Besut	Peninsular	0.181	0.050	0.201	-1.507	-1.377	0.112	0.038
Dungun	Peninsular	0.151	0.056	0.196	-1.723	-1.413	0.187	0.045
Hulu Terengganu	Peninsular	0.149	0.049	0.192	-1.745	-1.438	0.150	0.041
Kemaman	Peninsular	0.244	0.054	0.223	-1.128	-1.248	0.087	0.036
Kuala Terengganu	Peninsular	0.164	0.031	0.186	-1.630	-1.478	0.051	0.029
Marang	Peninsular	0.120	0.045	0.184	-1.989	-1.489	0.180	0.049
Setiu	Peninsular	0.000	0.000	0.204		-1.359		0.065

Model-based method: Using the selected model and adjusted for district-level covariate (proportion of Bumiputera ethnicity).
se.p.hat1: Design-based weighted standard errors of overweight prevalence.
p.hat1: Design-based weighted estimators of overweight prevalence.
p.hat2: Model-based weighted and smoothed estimators of overweight prevalence.
logit.p1: Design-based weighted estimators of logits of overweight prevalence.
logit.p2: Model-based weighted and smoothed estimators of logit of overweight prevalence.
logit.v1: Design-based weighted variances of logits of overweight prevalence.
logit.v2: Model-based weighted and smoothed variances of logits of overweight prevalence.
