# Supplementary figures and images for: A practical illustration of spatial smoothing methods for disconnected regions with INLA: spatial survey on overweight and obesity in Malaysia
Source: Int J Health Geogr. 2023 Jun 21;22:14. doi: 10.1186/s12942-023-00336-5 (PMC10286432; doi:10.1186/s12942-023-00336-5)

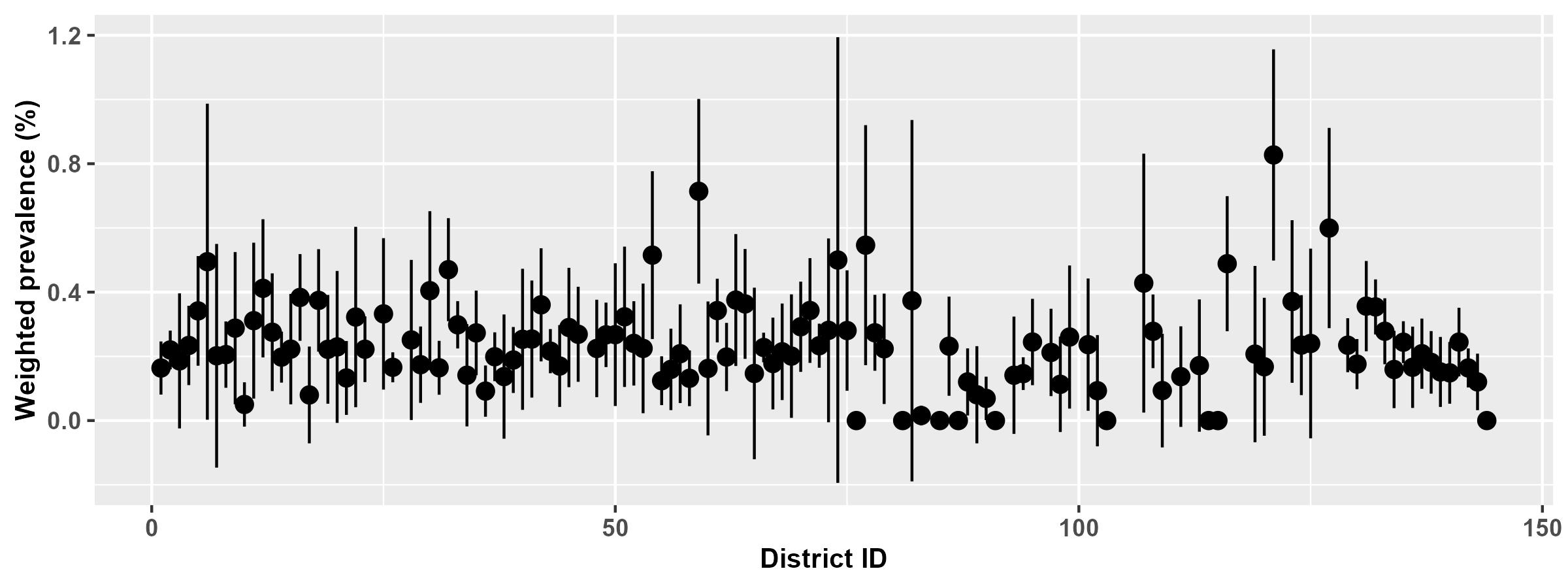

Supplement: Supplementary file 2 — Additional file 2: Figure S1. The uncertainty in the prevalence estimates of overweight shown in Fig. 2. Figure S2. The uncertainty in the respective estimated prevalence of overweight shown in Fig. 3. The top map corresponds to Model I, a single sum-to-zero constraint. The middle map corresponds to Model II, sum-to-zero constraints for each region and a common intercept. The bottom map corresponds to Model III, sum-to-zero constraint and intercept for each region. Figure S3. The uncertainty in the respective estimated prevalence of overweight shown in Fig. 4. The top map corresponds to Model IVa, split random effects with a common intercept. The middle map corresponds to Model IVb, split RE with separate intercepts. The bottom map corresponds to Model IVa adjusted for district-level covariate. [file 12942_2023_336_MOESM2_ESM.zip › Additional figure/Additional Figure 1.jpeg]

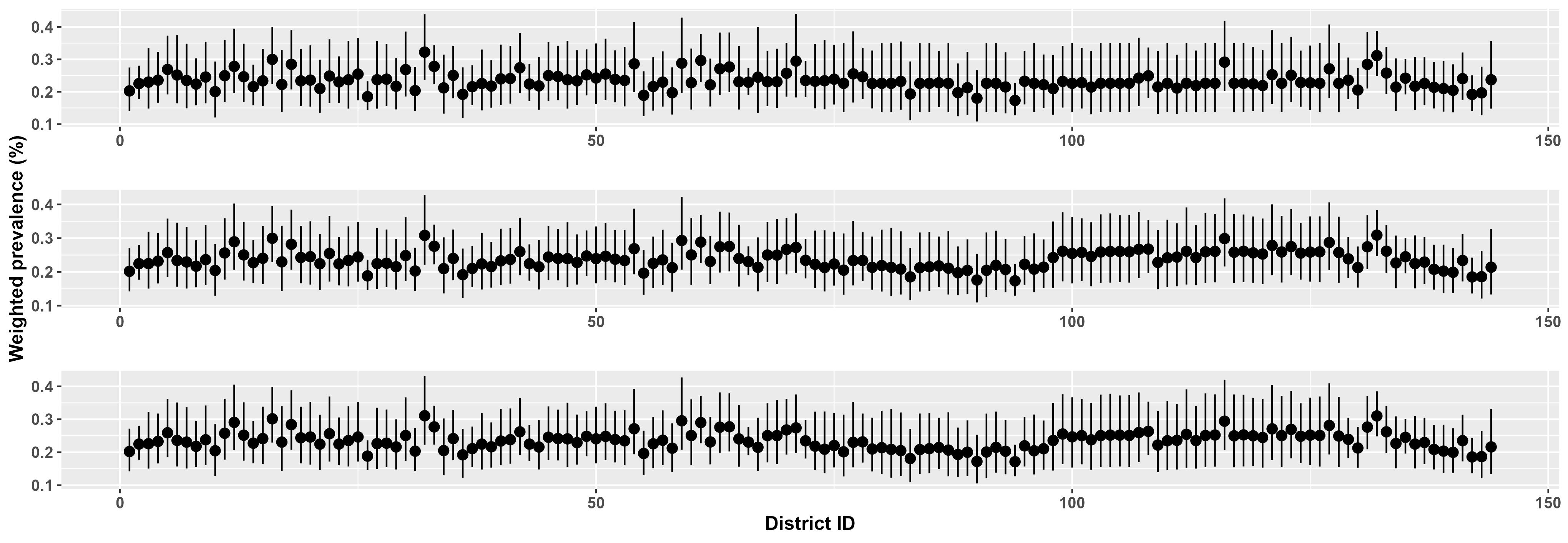

Supplement: Supplementary file 2 — Additional file 2: Figure S1. The uncertainty in the prevalence estimates of overweight shown in Fig. 2. Figure S2. The uncertainty in the respective estimated prevalence of overweight shown in Fig. 3. The top map corresponds to Model I, a single sum-to-zero constraint. The middle map corresponds to Model II, sum-to-zero constraints for each region and a common intercept. The bottom map corresponds to Model III, sum-to-zero constraint and intercept for each region. Figure S3. The uncertainty in the respective estimated prevalence of overweight shown in Fig. 4. The top map corresponds to Model IVa, split random effects with a common intercept. The middle map corresponds to Model IVb, split RE with separate intercepts. The bottom map corresponds to Model IVa adjusted for district-level covariate. [file 12942_2023_336_MOESM2_ESM.zip › Additional figure/Additional Figure 2.jpeg]

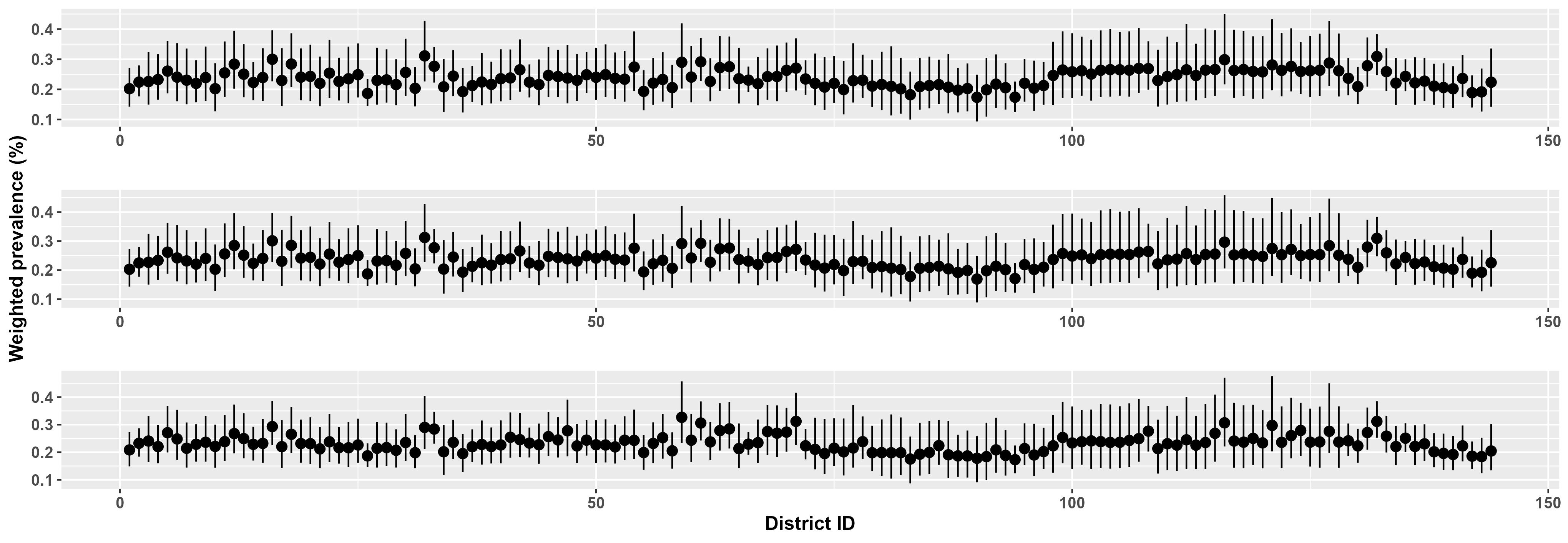

Supplement: Supplementary file 2 — Additional file 2: Figure S1. The uncertainty in the prevalence estimates of overweight shown in Fig. 2. Figure S2. The uncertainty in the respective estimated prevalence of overweight shown in Fig. 3. The top map corresponds to Model I, a single sum-to-zero constraint. The middle map corresponds to Model II, sum-to-zero constraints for each region and a common intercept. The bottom map corresponds to Model III, sum-to-zero constraint and intercept for each region. Figure S3. The uncertainty in the respective estimated prevalence of overweight shown in Fig. 4. The top map corresponds to Model IVa, split random effects with a common intercept. The middle map corresponds to Model IVb, split RE with separate intercepts. The bottom map corresponds to Model IVa adjusted for district-level covariate. [file 12942_2023_336_MOESM2_ESM.zip › Additional figure/Additional Figure 3.jpeg]
